# Multiphase Behavior of the Water + 1-Butanol + Deep Eutectic Solvent Systems at 101.3 kPa

**DOI:** 10.3390/molecules29204814

**Published:** 2024-10-11

**Authors:** Isadora Pires Gomes, Nicolas Pinheiro dos Santos, Pedro Bernardes Noronha, Ryan Ricardo Bitencourt Duarte, Henrique Pina Cardim, Erivaldo Antônio da Silva, Renivaldo José dos Santos, Leandro Ferreira-Pinto, Pedro Arce

**Affiliations:** 1 Department of Chemical Engineering, Lorena School of Engineering (EEL/USP), University of São Paulo, Lorena 12602-810, SP, Brazil; isadorapires@usp.br (I.P.G.); nicolas_santos@usp.br (N.P.d.S.); pedro_bernardes@usp.br (P.B.N.); ryan.ricardo@usp.br (R.R.B.D.); 2 Postgraduate Program in Science and Technology of Materials (POSMAT), School of Engineering and Sciences, São Paulo State University (UNESP), Rosana 19274-000, SP, Brazil; henrique.cardim@unesp.br (H.P.C.); renivaldo.santos@unesp.br (R.J.d.S.); 3 Department of Cartography, School of Science and Technology, São Paulo State University (UNESP), Presidente Prudente 19060-900, SP, Brazil; erivaldo.silva@unesp.br; 4 Department of Engineering, School of Engineering and Sciences, São Paulo State University (UNESP), Rosana 19274-000, SP, Brazil; leandro.f.pinto@unesp.br

**Keywords:** deep eutectic solvents, multiphase equilibria, experimental thermodynamics, 1-butanol, NRTL model

## Abstract

The growing demand for more sustainable routes and processes in the mixture separation and purification industry has generated a need to search for innovations, with new solvent alternatives being a possible solution. In this context, a new class of green solvents, known as deep eutectic solvents (DESs), has been gaining prominence in recent years in both academic and industrial spheres. These solvents, when compared to ionic liquids (ILs), are more environmentally friendly, less toxic, low-cost, and easier to synthesize. In addition, they have significantly lower melting points than their precursors, offering a promising option for various applications in this industrial sector. Understanding and studying the thermodynamic behavior of systems composed of these substances in purification and separation processes, such as liquid–liquid extraction and azeotropic distillation, is extremely important. This work aimed to study the phase behavior of liquid–liquid equilibrium (LLE) and vapor–liquid equilibrium (VLE) of water + 1-butanol + DES (choline chloride + glycerol) systems with a molar ratio of 1:2. Experimental LLE data, obtained at 298.15 K and 101.3 kPa, and VLE data, obtained at 101.3 kPa and in the temperature range of 364.05 K–373.85 K, were submitted to the thermodynamic quality/consistency test, proposed by Marcilla et al. and Wisniak, and subsequently modeled using the gamma–gamma approach for the LLE and gamma–phi for the VLE. The non-random two-liquid (NRTL) model was used to calculate the activity coefficient. The results are presented for the VLE in a temperature–composition phase diagram (triangular prism) and triangular phase diagrams showing the binodal curve and tie lines (LLE). The separation and distribution coefficients of LLE were determined to evaluate the extractive potential of the DES. For the VLE, the values of the relative volatility of the system were calculated, considering the entrainer free-basis, to evaluate the presence or absence of azeotropes in the range of collected points. From these data, it was possible to compare DES with ILs as extracting agents, using data from previous studies carried out by the research group. Therefore, the results indicate that the NRTL model is efficient at correlating the fluid behavior of both equilibria. Thus, this study serves as a basis for future studies related to the understanding and design of separation processes.

## 1. Introduction

The application of more environmentally sustainable solvents in the chemical industry is an emergent necessity due to the growing interest in combating the negative impact of this industrial sector on the ambient environment. In this context, the importance of diffusion in the use of this type of solvent in industry goes far beyond the chemical reaction area, embracing the separation and extraction processes of the mixture. Because large amounts of chemical residuals are produced by unit operations in this sector and the massive current use of highly toxic organic solvents, mainly obtained from oil, this issue is seen as a matter of important relevance [1].

In search of more sustainable alternatives, academic research has increasingly focused on new classes of solvents, known as “green” solvents. This class includes ionic liquids (ILs) and deep eutectic solvents (DESs). These solvents possess several advantages such as low toxicity, non-flammability, low volatility, rapid biodegradability, and recyclability. Owing to these attributes, green solvents can be effectively used in various production areas [2].

However, when comparing ILs and DES, DESs present significant advantages. The production of ILs involves complex synthesis and purification procedures, resulting in high cost and low biodegradability. In contrast, DESs stand out in chemical technology because of their specific properties that favor economic and environmental issues. With nearly zero toxicity, biodegradability, and economic production from natural reagents, DESs are particularly suitable for research in separation processes [3].

The most popular and studied type of deep eutectic solvent is choline chloride, which acts as a hydrogen bond acceptor (HBA). Most of these solvents are environmentally friendly organic compounds that retain the biological activity of the initial compounds and are safe for use in pharmaceutical, cosmetic, and food applications. Choline chloride is a low-cost and simple synthetic process with virtually no waste [4].

The production of DESs is straightforward and does not require complex labor processes; simply mixing and heating the components is sufficient. Additionally, they generally do not require further purification, because the purity of the final product depends on the purity of the initial components. Owing to their flexible physicochemical properties, DESs can be used in a wide range of applications. The properties of choline chloride-based DESs can be adjusted by varying the hydrogen bond donors, which allows the modulation of characteristics such as viscosity and polarity, making them highly adaptable to specific applications [5].

For the separation of azeotropic mixtures, DESs show promising capability to optimize processes related to aromatic–aliphatic and alcohol–hydrocarbon mixtures using the liquid–liquid extraction method. Alcohols are important industrial chemicals owing to their excellent physicochemical properties. The extraction of 1-butanol has received significant attention given its potential applications in various industrial sectors, including as a modern biofuel [6].

This study investigated the efficiency of 1-butanol extraction from water using a choline chloride-based DES, specifically utilizing glycerol as a hydrogen bond donor. DESs are noted for being economical and easy to prepare, making them energy-efficient and economically viable [3]. This study includes a comparative analysis to identify the specific dependencies and evaluate the performance of this DES in the extraction of 1-butanol.

## 2. Results and Discussions

### 2.1. Liquid–Liquid Equilibrium

#### 2.1.1. Binodal Curve and Liquid–Liquid Equilibrium Data

Detailed descriptions of the methodologies for obtaining binodal curves and tie lines have been presented in previous studies [7]. The experimental measurements for the ternary system were collected when the solution inside the equilibrium cell reached a constant turbidity point, after which the volumes were recorded and converted into compositions. The results for the DES and 1-butanol phases are listed in Table 1.

Binodal curve data were used to obtain the calibration curves. Table 2 presents the coefficients of Equation (1) for both phases.

After collecting the data for the binodal curve, the tie lines were determined and their compositions were determined using calibration curves (Table 2). To ensure the reliability of the data, the measurements were performed in triplicate, and the average values of the mass fractions found for each tie line are listed in Table 3.

From Table 4, in the 1-butanol phase, it is clear that the DES does not appear, indicating that only 1-butanol and water are present. It is also important to note that 1-butanol has limited miscibility in water. In ambient conditions, the solubility (weight percent) of n-butanol in water is 0.0650 (mass fraction), whereas that of water in n-butanol is 0.0224 at 323.15 K (mass fraction) [8]. Although the temperature in literature [8] is slightly different to the temperature in this work, the solubilities given by the literature are very similar to those found experimentally.

#### 2.1.2. Quality Tests

The compositions of the tie lines were subjected to a quality test. The overall mass balance deviation (δ) was calculated using Equation (6), resulting in an average deviation of 0.08% (Table 4).

Based on the results presented in Table 4, it can be concluded that the data exhibit good quality and can be used for thermodynamic modeling, as the individual deviations of each tie line adhere to the criterion of deviation of less than 0.2%.

#### 2.1.3. Separation Factor and Distribution Coefficients

The distribution coefficients and experimental separation factors were calculated using data validated by the quality test (Table 5).

A separation factor greater than one, S > 1, indicates the extraction potential of the DES for 1-butanol (solute) contained in water (diluent).

Based on the data from our research group, the separation factor values (S) for systems containing ionic liquids were used to compare the selectivity of DESs with ionic liquids (Table 6). The compared systems were {water + 1-butanol + [EMIM][EtSO_4_]} and {water + 1-butanol + [HMIM][BF_4_]}. The following average separation factor values were obtained for each system: 22.3456, 21.9875, and 13.0500 for [Ch+][Cl−]:Gly (1:2), [EMIM][EtSO_4_], and [HMIM][BF_4_] as extracting agents, respectively.

Analyzing the results and comparing how each solvent altered this parameter, it is evident that DES provides, on average, a higher separation factor in the removal of 1-butanol from aqueous solutions. Therefore, there is evidence that DES may be considered more effective extracting agents than both ionic liquids in terms of efficiency, as well as exhibiting higher biodegradability, easier synthesis, and lower cost.

#### 2.1.4. LLE Thermodynamic Modeling

In the thermodynamic modeling, the experimental LLE data were correlated using the γ-γ approach with the NRTL model to obtain the activity coefficients.

The binary interaction parameters for the NRTL model are presented in Table 7, and the non-randomness parameter values in the optimized system, $\alpha_{ij}$, were 0.2943, 0.843, and 0.3421 for the water, 1-butanol, and DES, respectively.

The absolute deviations for the compositions of both liquid phases, using the NRTL model with the γ-γ approach, are presented in Table 8.

**Figure 1:**
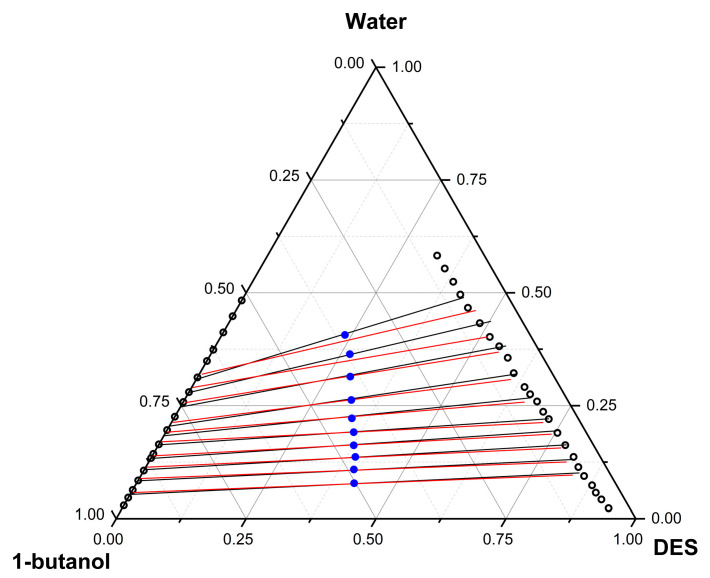
Phase diagram of the Water + 1-Butanol + DES system at 298.15 K (101.3 kPa).

The experimental data, binodal curve (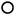), tie lines (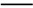), and thermodynamic modeling using the NRTL model (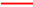) show a Type II diagram (Figure 1).

In Figure 1, the compositions of the solutions (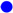) (Table 4) that produced the tie lines are also presented.

### 2.2. Vapor–Liquid Equilibrium

#### 2.2.1. Calibration Curves

The coefficients of the calibration curves and correlation coefficients (R^2^) obtained for the liquid phase through nonlinear second-order polynomial regression are shown in Table 9.

In this study, the DES was not considered to be present in the vapor phase, because of its extremely low vapor pressure [9]. Therefore, in contrast to the liquid phase, the vapor phase consisted of only two components: water and 1-butanol. Hence, only one calibration curve is required to fully correlate the experimental data, and in this work, it was used as the one related to density, since water and 1-butanol are not completely miscible and the refractive index measurements could have large deviations. The experimentally obtained expression for ρ versus the composition calibration curve is given by Equation (11).

$$\rho =-0.1297x_{1}^{3}+0.1651x_{1}^{2}+0.1524x_{1}+0.8181$$

#### 2.2.2. Vapor–Liquid Equilibrium Data

Experimental data for the bubble temperature, liquid, and vapor composition phases for the water (1) + 1-butanol (2) + DES (3) system VLE at 101.3 kPa, obtained through calibration curves, and calculated values for relative volatility are presented in Table 10.

To evaluate the capacity to improve the separation of the water + 1-butanol system in extractive distillation operations, it is possible to analyze the relative volatility of the system with the addition of a third component, an entrainer, which in this study was DES [Ch+][Cl−]:G (1:2); the greater than 1 are the obtained values for α_12_, better the entrainer’s separation effect is towards the system [10,11]. Because 6 of the 11 points collected experimentally had relative volatility values lower than 1, indicating that the system’s azeotrope has not been broken, the DES [Ch+][Cl−]:G (1:2) has not been considered an efficient entrainer to perform a possible extractive distillation of the water + 1-butanol + DES systems regarding the range of data analyzed.

#### 2.2.3. Thermodynamic Consistency

The results of the VLE experimental data and the results for the L-W test [12] regarding the consistency of the VLE experimental data are presented in Table 11. Additionally, the deviation value of the entire system was 1.77%.

Hence, in Table 11, the values for deviation were lower than 3%, and it is considered that the experimental data collected reached the required thermodynamic quality.

#### 2.2.4. VLE Thermodynamic Modeling

Thermodynamic modeling was applied to the experimental data using the Bubble T method associated with the gamma–phi approach, in which activity coefficients were calculated using the NRTL model. Optimized non-randomness parameter, $\alpha_{ij}$, and binary interaction parameters are shown in Table 12. Additionally, the calculated values for the bubble temperature and vapor composition, as well as the results, in terms of both variable percentage deviations, can be visualized in Table 13.

**Figure 2:**
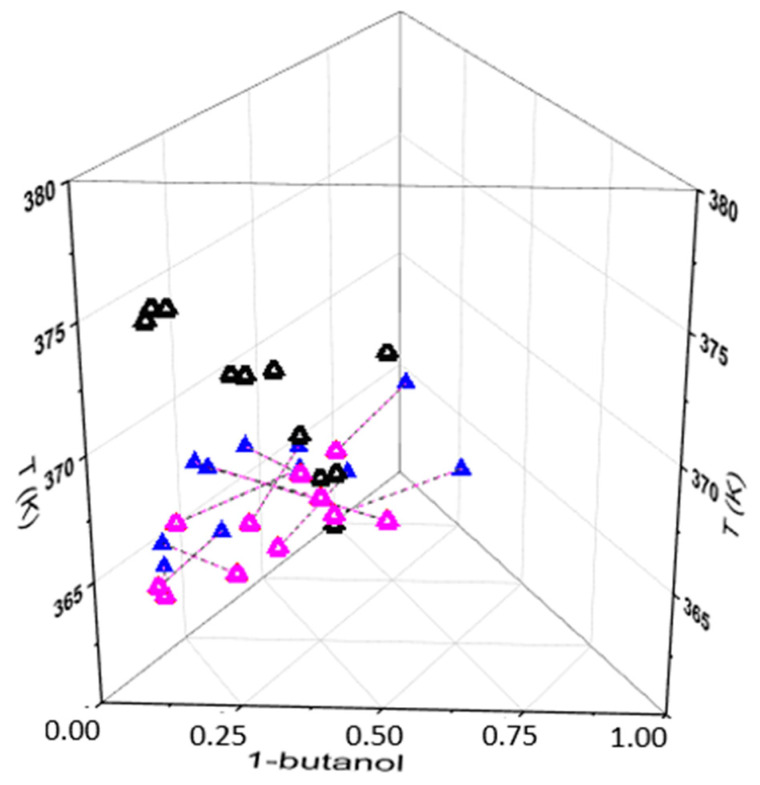
Ternary Txy phase diagram for experimental and NRTL model data for VLE of the water (1) + 1-butanol (2) + DES (3) systems (101.3 kPa).

Because all deviations obtained were lower than 5%, it was considered that the NRTL model could efficiently predict the VLE behavior of the water (1) + 1-butanol (2) + DES (3) systems, at 101.3 kPa. This statement can also be graphically visualized in a ternary Txy phase diagram (triangular prism), presented in Figure 2, in which the model-predicted data (**Δ**) and the liquid (**▲**) and vapor (**Δ**) experimental compositions are connected by VL lines (- - -).

## 3. Experimental

All the analytical and experimental methodologies utilized in this study have been previously published. Several research groups have discussed these techniques [13,14,15]. However, in the current work, only analytical and experimental methodologies are presented in minor detail.

### 3.1. Materials

The chemicals used in this study and their measured and previously reported physical properties, as well as their suppliers, are shown in Table 14. Measured values (Exp.) were compared with those reported in the literature. All solutions were prepared using an electronic balance (Qhaus Instruments Co., Ltd., Shanghai, China) with an accuracy of 0.0001 g. The densities and refractive indices were measured using a DMA 1001 densimeter with an accuracy of 0.0001 g/cm^3^ and an Abbemat 3200 refractometer with an accuracy of ±0.0001, both from Anton Paar, Graz, Austria.

### 3.2. Preparation of DES

To synthesize DES [Ch+][Cl−]:Gly (molar mass = 107.93 g.mol^−1^), choline chloride and glycerol were mixed in a molar ratio of 1:2. Using a heating magnetic agitator and temperature sensors, the phase reaction was conducted at a constant temperature (375 K) with stirring (200 rpm) until the mixture turned colorless, transparent, and homogeneous.

### 3.3. Experimental Equipment

**Figure 3:**
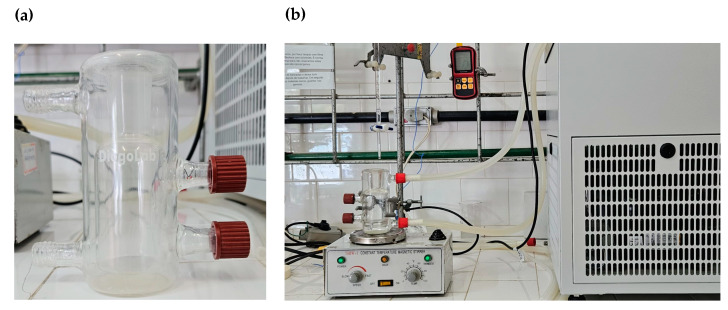
Experimental equipment used to collect LLE experimental data: (**a**) equilibrium cell; (**b**) all experimental equipment.

The experimental equipment for the LLE was composed of an equilibrium cell (volume of 300 cm^3^ and made of borosilicate), an ultrathermostatic bath (QUIMIS, Diadema, SP, Brazil, Q214m2) (to control the temperature in the equilibrium cell), a magnetic stirrer (for stirring the liquid inside the equilibrium cell, SPPENCER, Easton, PA, USA, model SP108-25), and thermometers (Kangaroo Traceable, model GM1312, accuracy of ±0.1 K, Hampton, NH, USA) (Figure 3). The equilibrium cell (Figure 3a) has openings protected by septa, is used to take samples of the liquid phases, and has an input and output flow attached to the ultrathermostatic bath, and it is open at the top, where the substances are added and the temperature of the solution is taken.

**Figure 4:**
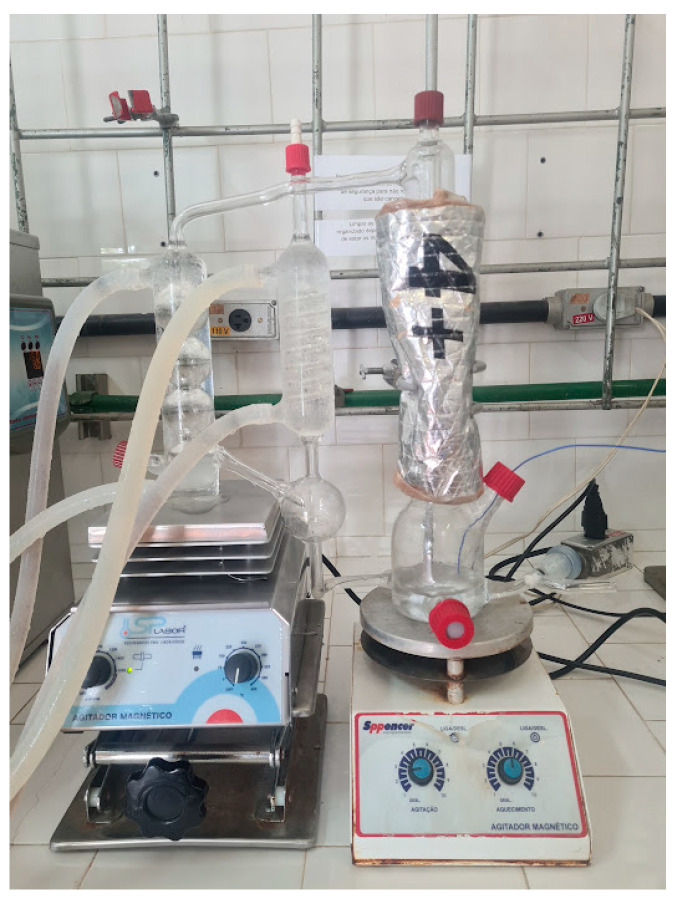
Experimental equipment used to collect VLE experimental data.

The experimental equipment utilized to collect vapor–liquid equilibrium data can be visualized as shown in Figure 4. Similar to the one described by [19], it is composed of an equilibrium cell; a modified Othmer ebulliometer (equipment’s main device, fabricated with borosilicate glass), which is associated with an ultrathermostatic bath (QUIMIS, Diadema, SP, Brazil, Q214m2 Q214m2), which controls the cooling water temperature that circulates through the ebulliometer; a liquid phase digital thermometer (Kangaroo Traceable, model GM1312, accuracy of ±0.1 K, Hampton, NH, USA); one magnetic heater; a stirrer for the liquid phase (SPPENCER, model SP108-25) to provide heat and maintain homogeneity; and one magnetic stirrer for the vapor phase (SP LABOR, Presidente Prudente, SP, Brazil, model SP- 160), allowing more precise experimental measures regarding this phase since water and 1-butanol are not completely miscible.

### 3.4. Binodal Curve and Liquid–Liquid Equilibrium

To evaluate the separation efficiency of the 1-butanol−water system, a study was conducted on the liquid–liquid equilibrium in pseudoternary systems of 1-butanol−water–DES at 298.15 K and atmospheric pressure. In this study, DES was considered a pseudocomponent to follow the phase behavior patterns of the mixtures studied.

The binodal curve of the pseudo-ternary systems was determined by preparing various pseudo-binary mixtures of water–DES in the immiscible region, and 1-butanol was added until the pseudo-ternary mixture reached the cloud point.

The analysis of tie lines was carried out using solutions with different volumes of water, 1-butanol, and DES inserted into the cell under magnetic stirring for approximately 30 min and then left to stand until phase equilibrium occurred. The attainment of phase equilibrium was indicated by the transparency of both phases in the system. Liquid–liquid equilibrium was reached in 6 h in systems at 298.15 K. Quantitative analysis of the phases was performed by sampling and analysis using the refractive index (nD) and density (ρ). Each sample was analyzed three times to ensure data reliability

### 3.5. Calibration Curves

Calibration curves and correlation coefficients (R^2^) were obtained from the binodal curve data using the refractive indices and densities for each point. A nonlinear regression was performed using OriginPro 2024 software, applying a second-order polynomial, as represented by Equation (1):

(1) $$F(w_{1},w_{2})=A+B\;*\;w_{1}+C\;*\;w_{2}+D\;*\;{w_{1}}^{2}+E\;*\;w_{1}\;*\;w_{2}+F\;*\;{w_{2}}^{2}$$

where *F*(*w*_1_, *w*_2_) represents the refractive index (nD) or the density (ρ).

This methodology was then employed to obtain the tie lines for the LLE and the compositions of the VLE.

### 3.6. Vapor–Liquid Equilibrium Experimental Data

To construct calibration curves for the liquid and vapor phases (Equation (1)), nD and ρ were measured for a large number of different solutions of known composition.

Then, the experimental vapor–liquid equilibrium data were collected using the experimental equipment (Figure 4). Throughout this process, it was considered that the system reached the thermodynamic stage of equilibrium at the moment both the number of condensed vapor drops and the temperature had been stated as constant over time. Then, both the liquid and vapor phases were collected, their respective densities and refractive indices were measured, and it was possible to obtain the composition values for the two phases.

### 3.7. Thermodynamics Consistency and Quality Test

The VLE L–W test [12] was used to evaluate the consistency of the experimentally collected data regarding the vapor–liquid equilibrium. In this test, two parameters, L and W, were calculated from the experimental data and should be as close as possible. Hence, the results are presented as a percentage deviation, which is considered acceptable if lower than 3%. The mathematical description of this consistency test is represented by Equations (2)–(4).

(2) $$W_{i}=\frac{RT}{∆S}[\sum x_{k}\ln(\gamma_{k})-\sum x_{k}\ln(\frac{y_{k}}{x_{k}})]$$

(3) $$L_{i}=\frac{\sum T_{k}^{SAT}x_{k}{∆S}_{k}^{SAT}}{\sum x_{k}{∆S}_{k}^{SAT}}-T$$

(4) $$D=\frac{\left|L-W\right|}{L+W}100\%$$

Regarding the liquid–liquid equilibrium experiments, the quality was assessed by comparing the sum of the calculated mass in both phases with the actual total mass used, as described by [20]. An overall mass balance deviation (δ) below 0.5% ensures the data quality. The mass balance of each component in the ternary system was calculated using Equation (5):

(5) $$\begin{array}{c} M^{sol}x_{1}^{sol}=M^{L1}x_{1}^{L1}+M^{L2}x_{1}^{L2}\\ M^{sol}x_{2}^{sol}=M^{L1}x_{2}^{L1}+M^{L2}x_{2}^{L2}\\ M^{sol}x_{3}^{sol}=M^{L1}x_{3}^{L1}+M^{L2}x_{3}^{L2} \end{array}\;\;\;\;\;\;\;\;\;\;\;\;\;\;\;\;\;\;\;\underset{\underset{M}{⎵}}{\left[\begin{array}{c} M^{sol}x_{1}^{sol}\\ M^{sol}x_{2}^{sol}\\ M^{sol}x_{3}^{sol} \end{array}\right]}=\underset{\underset{B}{⎵}}{\left[\begin{array}{c} x_{1}^{L1}\\ x_{2}^{L1}\\ x_{3}^{L1} \end{array}\;\;\begin{array}{c} x_{1}^{L2}\\ x_{2}^{L2}\\ x_{3}^{L2} \end{array}\right]}\cdot \underset{\underset{P}{⎵}}{\left[\begin{array}{c} M^{L1}\\ M^{L2} \end{array}\right]}$$

The vector P is represented by $P=X^{-1}B^{T}M,$ where B^T^ represents the transpose of matrix B and X^−1^ is the inverse of X $=\left(B^{T}B\right)$. From P, m^L1^ and m^L2^ were obtained by comparing the sum with the value of m^sol^. The overall mass-balance deviation can be obtained as follows:

(6) $$\delta \left(\%\right)=100\cdot \frac{\left|\left(M^{L1}+M^{L2}\right)-M^{sol}\right|}{M^{sol}}$$

### 3.8. Separation Factor, Distribution Coefficient, and Relative Volatility

The separation factor (S) and distribution coefficient (D_i_) were calculated to evaluate the solvent extraction capability in the liquid–liquid extraction processes. The coefficients D_1_ and D_2_ are determined from the tie lines of the system using molar or mass fractions and are defined as D_1_ = (x_1_)^phase 3^/(x_1_)^phase 1^ and D_2_ = (x_2_)^phase 3^/(x_2_)^phase 1^, where 1, 2, and 3 represent the diluent, solute, and solvent, respectively, and S = D_2_/D_1_ [21].

To assess the performance as an entrainer in the potential extractive distillation of the water + 1-butanol system, the relative volatility parameter was used, calculated from the experimental VLE data. This parameter was calculated similarly to the method employed in the literature [22], $\alpha_{ij}={(y}_{i}/x_{i})/{(y}_{j}/x_{j})$, where i is the most volatile component compared to other components j, and $y_{i,j}$ and $x_{i,j}$ are the vapor and liquid phase compositions of the experimental components i and j, respectively.

### 3.9. Modeling and Computation

#### 3.9.1. Gibbs Free Energy Minimization

Calculations were performed based on the Gibbs free energy minimization [23]. The total Gibbs free energy of a mixture of n components and π phases is obtained from the following equations:

(7) $$G=\sum_{k=1}^{\pi }n^{k}g^{k}=\sum_{k=1}^{\pi }\left(\sum_{i=1}^{n}n_{i}^{k}\right)g^{k}$$

where n^k^ and n^k^_i_ are the total number of moles in phase k and the number of moles of component i in phase k, respectively.

In the case of using the Gibbs energy functions for the LLE calculations, the following equation is utilized to calculate the Gibbs energy of the mixture:

(8) $$G=\sum_{k=1}^{\pi }\sum_{i=1}^{n}n_{i}^{L(k)}\left[ln\left(x_{i}^{L(k)}\gamma_{i}^{L(k)}\right)\right]$$

where the superscript L(k) denotes the liquid phase k, and γ_i_ represents the activity coefficient of component i. Through the minimization of the above equation, equilibrium concentrations are calculated in each phase.

#### 3.9.2. Liquid–Liquid Equilibrium

Thermodynamic modeling of liquid–liquid equilibrium was based on the conditions of uniform pressure, temperature, and fugacity of both liquid phases at equilibrium. Considering the pressure and temperature constants, the gamma–gamma approach was used, as shown in Equation (9).

(9) $$x_{i}^{L1}\gamma_{i}^{L1}=x_{i}^{L2}\gamma_{i}^{L2}$$

#### 3.9.3. Vapor–Liquid Equilibrium

Because vapor–liquid equilibrium experiments were conducted at low pressures (101.3 kPa), the chosen approach to represent the system thermodynamic behavior in this work was gamma–phi [24], also known as the Modified Raoult’s law, described in Equation (10).

(10) $$x_{i}\gamma_{i}P_{i}^{SAT}\phi_{i}^{SAT}{POY}_{i}=y_{i}P{\hat{\emptyset }}_{i}^{V}\overset{Low\;pressures}{\Rightarrow }x_{i}\gamma_{i}P_{i}^{SAT}=y_{i}P$$

where POY_i_ is the Poynting factor and $P_{i}^{SAT}$ represents the saturation vapor pressure of the pure component *i*.

This approach consists of the principle of isofugacity between the liquid and vapor phases, in which the activity coefficient is the main representative of the ideality deviation. Additionally, the Bubble T calculation method [23] was applied to this approach, which enabled the attainment of bubble temperature and vapor phase composition values through many iterations with fixed values for the system pressure and liquid phase composition.

#### 3.9.4. Non-Random Two-Liquid Model (NRTL)

The activity coefficients, g_i_, were calculated using the NRTL model [25] based on the local composition concept, and they are applicable to partial miscibility systems. The multicomponent shape of this model is summarized in Equations (11) and (12).

(11) $$\ln\gamma_{i}=\frac{\sum_{j}\tau_{ji}G_{ji}x_{j}}{\sum_{k}G_{ki}x_{k}}+\sum_{j}\frac{x_{j}G_{ij}}{\sum_{k}G_{kj}x_{k}}\left(\tau_{ij}-\frac{\sum_{r}x_{r}\tau_{rj}G_{rj}}{\sum_{k}G_{kj}x_{k}}\right)$$

(12) $$\tau_{ji}=\frac{g_{ji}-g_{ii}}{RT}\;;\;\;\;G_{ji}=\exp\left(-\alpha_{ji}\tau_{ji}\right)\;;\;\;\;(\alpha_{ji}=\alpha_{ij})$$

where $\tau_{ij}$ are the characteristic energy parameters of i-j molecule interactions and $\alpha_{ij}$ is the non-randomness parameter of the mixture, i.e., its components are distributed along a pattern dictated by local composition. If the $\alpha_{ij}$ value is equal to zero, the mixture is considered completely random.

## 4. Conclusions

In this study, the thermodynamic behavior of the liquid–liquid and vapor-liquid phase equilibria of {water + 1-butanol + choline chloride/glycerol (1:2)} systems was studied at 101.3 kPa. Using the described experimental equipment, data were collected from experiments to construct binodal curves and tie lines (LLE) and to obtain bubble temperatures and vapor phase compositions (VLE). The data were subsequently validated using quality and thermodynamic consistency tests to obtain consistent experimental data. Thermodynamic modeling was then carried out using the gamma–gamma (LLE) and gamma–phi approaches employing the Bubble T method (VLE), with the NRTL model used to calculate activity coefficients. Based on the results obtained from modeling and the low calculated deviation values, as well as graphical analyses through ternary diagrams and triangular prisms, it was observed that the NRTL model efficiently represented the fluid phase behavior of the water + 1-butanol + DES systems in the LLE and VLE. Finally, the distribution and separation coefficients as well as the relative volatility parameter values were calculated from the experimental LLE and VLE data, respectively. Since all values found for the distribution and separation coefficients were greater than 1, it was concluded that DES: [Ch+][Cl−] + Gly (1:2, molar ratio) showed potential for use as an effective extraction agent, making it suitable for future studies involving separation operations and extraction. However, because half of the calculated values for relative volatility were lower than 1.0, [Ch+][Cl−] + Gly was not considered an effective entrainer in possible extractive distillation operations according to the range of data analyzed.

## Figures and Tables

**Table 1:** Experimental LLE binodal curve of the water (1) + 1-butanol (2) + DES (3) system at 298.15 K and P = 101.3 kPa (w_1_, w_2_, and w_3_—mass fractions).

DES Phase			1-Butanol Phase		
w_1_	w_2_	w_3_	w_1_	w_2_	w_3_
0.0231	0.0408	0.9361	0.0293	0.9707	0.0001
0.0574	0.0483	0.8943	0.0634	0.9366	0.0004
0.0737	0.0473	0.8790	0.0845	0.9155	0.0001
0.0943	0.0523	0.8534	0.1063	0.8937	0.0002
0.1624	0.0544	0.7832	0.1643	0.8357	0.0001
0.2203	0.0574	0.7224	0.2256	0.7744	0.0003
0.2365	0.0599	0.7036	0.2583	0.7417	0.0001
0.2593	0.0603	0.6804	0.2802	0.7198	0.0002
0.2754	0.0657	0.6589	0.3127	0.6873	0.0004
0.3738	0.6262	0.0001	0.3223	0.0738	0.6039
0.4482	0.5518	0.0003	0.3818	0.0719	0.5463

**Table 2:** Coefficients of the calibration curves: nD versus composition and ρ versus composition.

Coefficients	DES Phase		1-Butanol Phase	
	nD	ρ (g.cm^−3^)	nD	ρ (g.cm^−3^)
A	1.4953	1.1317	1.3989	0.8114
B	−0.2949	−0.4582	−0.0683	0.1288
C	−0.3697	2.0295	1.0000	1.0000
D	0.1368	0.0805	0.0066	0.1030
E	1.0653	4.0021	1.0000	1.0000
F	−0.7060	−27.7646	1.0000	1.0000
R^2^	0.9956	0.9982	0.9987	0.9957

**Table 3:** Tie lines of the water (1) + 1-butanol (2) + DES (3) system at 298.15 K and P = 101.3 kPa.

1-Butanol Phase			DES Phase		
w_1_	w_2_	w_3_	w_1_	w_2_	w_3_
0.0534	0.9466	0.0000	0.1009	0.0589	0.8402
0.0836	0.9164	0.0000	0.1306	0.0585	0.8109
0.1082	0.8918	0.0000	0.1631	0.0523	0.7846
0.1329	0.8671	0.0000	0.1942	0.0548	0.7510
0.1630	0.8370	0.0000	0.2210	0.0571	0.7219
0.1834	0.8166	0.0000	0.2674	0.0685	0.6641
0.2047	0.7953	0.0000	0.3203	0.0686	0.6112
0.2468	0.7532	0.0000	0.3821	0.0592	0.5587

**Table 4:** Quality test of the water (1) + 1-butanol (2) + DES (3) system at 298.15 K and P = 101.3 kPa.

Solution				M^BUT^	M^DES^	M^sol^	δ
M^sol^	w_1_	w_2_	w_3_				
7.9456	0.0783	0.5028	0.4189	3.9744	3.9628	7.9373	0.1047
9.0453	0.1087	0.4881	0.4032	4.5321	4.5007	9.0328	0.1380
8.9321	0.1364	0.4715	0.3921	4.4616	4.4656	8.9272	0.0551
7.9536	0.1622	0.4617	0.3761	3.9815	3.9793	7.9608	0.0901
6.9324	0.1912	0.4473	0.3615	3.4666	3.4689	6.9355	0.0451
8.0435	0.2221	0.4353	0.3426	3.9309	4.1277	8.0586	0.1881
6.9834	0.2625	0.4163	0.3212	3.3343	3.6540	6.9883	0.0700
7.4537	0.3145	0.3924	0.2932	3.5678	3.8864	7.4542	0.0067

**Table 5:** Separation factors and distribution coefficients for 1-butanol.

S	D_1_	D_2_
30.3669	0.0622	1.8895
24.4718	0.0638	1.5622
25.7035	0.0586	1.5074
23.1291	0.0632	1.4615
19.8638	0.0682	1.3556
17.3812	0.0839	1.4580
18.1513	0.0862	1.5645
19.6979	0.0786	1.5482

**Table 6:** Comparison of separation factors for systems containing DES and ILs.

ChCl:Gly	[EMIM][EtSO_4_]	[HMIM][BF_4_]
30.37	3.82	2.01
24.48	6.91	3.63
25.70	16.60	5.64
23.13	17.54	7.42
19.86	22.82	11.62
17.38	24.71	16.30
18,15	13.81	23.11
19.70	23.83	24.81

**Table 7:** NRTL binary interaction parameters, Δg_ij_ = g_ji_ − g_ii_ (cal.mol^−1^), for the LLE of the water (1) + 1-butanol (2) + DES (3) systems at 298.15 K and P = 101.3 kPa.

Component	1	2	3
1	0.0000	−15.4567	−5.4351
2	350.0342	0.0000	1805.5474
3	580.9543	657.4356	0.0000

**Table 8:** Absolute deviations for the water (1) + 1-butanol (2) + DES (3) systems at 298.15 K and P = 101.3 kPa.

1-Butanol Phase						DES Phase					
w_1_	w_2_	w_3_	Δw_1_%	Δw_2_%	Δw_3_%	w_1_	w_2_	w_3_	Δw_1_%	Δw_2_%	Δw_3_%
0.0576	0.9386	0.0038	0.4200	0.8000	0.3800	0.0963	0.0735	0.8301	0.4600	1.4600	1.0100
0.0884	0.9096	0.0020	0.4800	0.6800	0.2000	0.1252	0.0711	0.8036	0.5400	1.2600	0.7300
0.1138	0.8849	0.0014	0.5600	0.6900	0.1400	0.1564	0.0662	0.7774	0.6700	1.3900	0.7200
0.1390	0.8583	0.0027	0.6100	0.8800	0.2700	0.1867	0.0694	0.7438	0.7500	1.4700	0.7200
0.1699	0.8281	0.0020	0.6900	0.8900	0.2000	0.2126	0.0723	0.7151	0.8400	1.5200	0.6800
0.1911	0.8052	0.0037	0.7700	1.1400	0.3700	0.2578	0.0858	0.6564	0.9600	1.7300	0.7700
0.2129	0.7839	0.0031	0.8200	1.1400	0.3100	0.3078	0.0870	0.6051	1.2400	1.8500	0.6100
0.2567	0.7388	0.0045	0.9900	1.4400	0.4500	0.3686	0.0800	0.5514	1.3500	2.0800	0.7300

**Table 9:** Calibration curves: nD versus composition and ρ versus composition and correlation coefficients for the liquid phase.

Coefficient	nD	ρ (g.cm^−3^)
A	1.3948	0.7881
B	−0.0205	0.2056
C	−0.0131	0.4488
D	−0.0489	0.0000
E	0.0247	−0.0551
F	0.0968	−0.1809
R^2^	0.9879	0.9917

**Table 10:** Experimental values of the VLE composition phases (mass fractions) and bubble temperatures for the water (1) + 1-butanol (2) + DES (3) system (P = 101.3 kPa).

T (K)	Liquid Phase			Vapor Phase		α_12_
	w_1_	w_2_	w_3_	w_1_	w_2_	
364.05	0.3198	0.3825	0.2977	0.5776	0.4225	1.6351
365.55	0.6851	0.2052	0.1097	0.6377	0.3623	0.5272
365.85	0.7506	0.1382	0.1112	0.0000	0.0000	0.2814
366.25	0.2683	0.5329	0.1988	0.5820	0.4180	2.7655
366.55	0.5404	0.2306	0.2290	0.8526	0.1474	2.4683
366.75	0.5012	0.1757	0.3232	0.7266	0.2734	0.9317
367.25	0.4174	0.2695	0.3131	0.6786	0.3214	1.3632
368.05	0.8360	0.0517	0.1124	0.7509	0.2492	0.1863
368.75	0.6851	0.0897	0.2252	0.8880	0.1120	1.0381
372.05	0.8335	0.0509	0.1156	0.8771	0.1230	0.4355
373.85	0.7291	0.0471	0.2238	0.4913	0.5087	0.0624

**Table 11:** L-W test results of the VLE experimental data.

L	W	D (%)	Test Result
43.6660	41.7587	2.2327	Consistent
15.4448	15.3963	0.1570	Consistent
13.0551	13.0713	0.0621	Consistent
42.0124	39.8682	2.6187	Consistent
26.2802	25.6792	1.1567	Consistent
31.1506	30.2904	1.4000	Consistent
37.2356	35.7064	2.0964	Consistent
8.4157	8.4684	0.3119	Consistent
16.7547	16.6136	0.4229	Consistent
7.7959	7.7745	0.1375	Consistent
18.5737	18.0475	1.4370	Consistent

**Table 12:** NRTL VLE optimized non-randomness parameters, $\alpha_{ij}$, and binary interaction parameters, $\Delta g_{ij}=g_{ji}-g_{ii}\;$(cal.mol^−1^), for the water (1) + 1-butanol (2) + DES (3) systems.

Component	1	2	3	$\alpha_{ij}$
1	0.0000	9.3969	1.9093	0.3925
2	0.1520	0.0000	49.6237	0.2939
3	2.7052	8.6696	0.0000	0.1243

**Table 13:** The bubble temperature and vapor composition (mass fraction) obtained using the NRTL model and their respective deviations (%) for the VLE of the water (1) + 1-butanol (2) + DES (3) system (P = 101.3 kPa).

T (K)	$w_{1}$	$w_{2}$	ΔT	$\Delta w_{1}$	$\Delta w_{2}$
369.48	0.6275	0.3725	1.14	3.80	3.79
370.91	0.6877	0.3123	0.24	1.79	1.78
369.31	0.6545	0.3455	0.16	4.28	4.28
367.50	0.6320	0.3680	0.00	5.00	5.00
375.54	0.8793	0.1207	2.65	4.98	4.97
373.14	0.6766	0.3234	2.52	1.53	1.52
373.34	0.7286	0.2714	2.22	5.00	5.00
373.14	0.7648	0.2352	2.89	4.98	4.92
375.08	0.8380	0.1620	3.27	3.11	3.25
375.55	0.9143	0.0857	3.73	4.98	4.95
374.06	0.5413	0.4587	2.19	4.90	4.89
Average deviation:			1.91	4.04	4.02

**Table 14:** Properties of the compounds investigated in this study (298.15 K).

Compound	Supplier	Purity (%)	Experimental		Literature	
			nD	ρ (g.cm^−3^)	nD	ρ (g.cm^−3^)
1-Butanol	Synth	≥99.8	1.3985	0.8006	1.3973 [16]	0.8060 [16]
Water	-	Bi-distilled	1.3337	0.9989	1.3325 [17]	0.9970 [17]
[Ch+][Cl−]: Glycerol	-	≈100.0	1.4812	1.1912	1.4868 [18]	1.1913 [18]

Experimental uncertainties: u(T) = 0.1 K, u(P) = 0.1 kPa, u(ρ) = 0.0001 g.cm^−3^ and u(nD) = 0.0001.

## Data Availability

The original contributions presented in this study are included in the article and further inquiries can be directed to the corresponding authors.
